# Coassembly of a Hybrid Synthetic–Biological Chitosan-*g*-Poly(*N*-isopropylacrylamide) Copolymer with DNAs of Different Lengths

**DOI:** 10.3390/polym16213101

**Published:** 2024-11-04

**Authors:** Maria Karayianni, Elena-Daniela Lotos, Marcela Mihai, Stergios Pispas

**Affiliations:** 1 Petru Poni Institute of Macromolecular Chemistry, 41A Grigore Ghica Voda Alley, 700487 Iasi, Romania; m.karayianni@icmpp.ro (M.K.); daniela.lotos@icmpp.ro (E.-D.L.); 2 Theoretical and Physical Chemistry Institute, National Hellenic Research Foundation, 48 Vassileos Constantinou Ave., 116 35 Athens, Greece

**Keywords:** chitosan, poly(*N*-isopropylamide), graft copolymers, DNA, electrostatic interactions, gene delivery, thermoresponsiveness

## Abstract

Natural polysaccharides can serve as carriers of genes owing to their intrinsic biocompatibility, biodegradability, and low toxicity. Additionally, they can be easily chemically modified, e.g., through grafting, leading to hybrid synthetic–biological copolymers with additional functionalities. In this work we report on the electrostatic interaction between a chitosan-*g*-poly(*N*-isopropylacrylamide) (Chit-*g*-PNIPAM) copolymer and DNA macromolecules of different lengths (i.e., 50 and 2000 bp), towards the construction of polyplexes that can serve as potential gene delivery systems. At the basic science level, the work aims to elucidate the effects of DNA length on the structural and physicochemical properties of the thermoresponsive hybrid macromolecular assemblies. The protonated amino groups on the chitosan backbone enable electrostatic binding with the anionic phosphate groups of the DNA molecules, while the PNIPAM side chains are expected to impart thermoresponsive properties to the formed polyplexes. Different amino to phosphate group (N/P) mixing ratios were examined, aiming to produce stable dispersions. The physicochemical properties of the resulting polyplexes were investigated by dynamic and electrophoretic light scattering (DLS and ELS), while their morphology was studied by scanning-transmission electron microscopy (STEM). Moreover, their response to changes in temperature and ionic strength, as well as their stability against biological media, was also examined. Finally, the binding affinity of the copolymer towards DNA was evaluated through fluorescence spectroscopy, using ethidium bromide quenching assays, while infrared spectroscopy was used to investigate the structure of the incorporated DNA chains.

## 1. Introduction

In recent years numerous biotechnological applications based on drug delivery and gene therapy have emerged as promising therapeutic approaches against various diseases, including malignant tumors, cardiovascular diseases, infections, neurodegenerative disorders, as well as several genetic conditions [1]. Nevertheless, there are still some challenges to overcome related to poor pharmacokinetics, non-targeted delivery, rapid degradation, unsatisfactory efficacy, and side effects upon the introduction of nucleic acids, proteins, peptides, or other substances into body circulation [1]. Therefore, for the successful delivery of drugs and genes, suitable carriers are needed, viral vectors being the most commonly used ones, especially for large-scale applications, although they have certain disadvantages such as immune response, limited payload capacity, insertional mutagenesis, and pre-existing immunity [2]. On the other hand, non-viral systems mostly based on polymers, liposomes, and nanoparticles offer lower immunogenicity, increased safety, no size limitation of the molecules to be introduced, low cost of production, and ease of manufacturing [1,2]. Particularly, polymeric carriers are being extensively studied since they offer essential advantages like enhanced stability, protection from nuclease degradation, and facilitation of cellular entry [3]. Among the plethora of available polymers of either biological or synthetic origin, natural polysaccharides show great potential to be utilized as non-viral vectors owing to their intrinsic biocompatibility, biodegradability, low toxicity, and cationic charge [4,5,6,7,8,9,10,11].

Chitosan is produced by the acetylation of chitin, which constitutes one of the most abundant biopolymers in nature since it can be isolated from the exoskeleton of marine crustaceans, such as crabs and shrimps, the cuticles of insects, and the cell walls of micro-organisms and fungi [4,6,7]. It is essentially a linear polysaccharide comprising *D*-glucosamine and *N*-acetyl glucosamine alternating repeating units, with the repetition of the units being determined by the degree of deacetylation (DD) [6]. Unsurprisingly, it has been extensively used in numerous bioapplications concerning drug and/or gene delivery systems, owing to its innate advantageous properties, which include inherent biocompatibility, biodegradability, and low toxicity, as well as verified antimicrobial and mucoadhesive properties [3,5,11]. Chitosan has a pK_a_ dissociation constant around 6.5 due to the presence of the amino groups, which makes it rather insoluble in water but readily soluble in acidic media, where it is positively charged due to the protonation of its primary amine groups [4,6]. Positively charged polymers exhibit great potential as gene delivery vehicles since they can easily bind the negatively charged genetic material, thus forming stable complexes known as polyplexes. Additionally, as the cellular and nuclear membranes are negatively charged, their interaction with the positively charged chitosan facilitates the cellular uptake of the corresponding polyplexes and the transport of the cargo into the nucleus of the cell [11]. Another advantage of the positive charge of chitosan is the fact that it renders it capable of endosomal escape due to the ability of the amino groups to capture protons, a phenomenon known as the “proton-sponge effect” [3,11]. In brief, the chitosan amino groups become protonated in the acidic environment inside the endosome, which in turn causes the accumulation of water and chloride ions from the endoplasm, eventually leading to endosome rupture. Lastly, the presence of both amino and hydroxyl functional groups enables the chemical modification of chitosan through various types of reactions, resulting to polymers with altered characteristics and behavior [7,8,9]. According to the specific reaction conditions, processes such as etherification, esterification, crosslinking, graft copolymerization, and *O*-acetylation can be performed on the hydroxyl groups, whereas reactions like acetylation, quaternization, Schiff’s base reaction, and grafting occur on the amino groups [7].

Chemical modification of chitosan is a well-established and widely used method of overcoming any possible drawbacks or limitations, thus improving the performance of potential chitosan carriers, since it can enhance its solubility in aqueous media, rheological characteristics, stability towards heat and temperature, and oxidation resistance [7]. Among the various chemical modification strategies and approaches, the grafting of chitosan with monomers and polymers of either natural or chemical origin serves as a crucial method for the functionalization and application of chitosan [8]. The incorporation of polymers onto the polysaccharide backbone imparts chitosan with enhanced adhesive properties and water solubility, as well as other desirable attributes [8]. Several derivatives produced in this way have been applied for therapeutic and pharmaceutical usages related to orthopedics, wound healing, engineering of specific tissues, as well as precise drug or gene delivery to particular body parts [7]. As can be expected, there are several experimental studies investigating the capacity of different grafted chitosan derivatives to be utilized as effective gene or drug delivery vehicles [12,13,14,15,16,17,18,19]. One of the most intriguing prospects of chitosan grafting is the opportunity to impart additional functionalities, like responsiveness to external stimuli, by choosing appropriate grafted copolymers. The use of the renowned temperature-responsive poly(*N*-isopropylacrylamide) (PNIPAM) is one such exemplary case that has attracted considerable scientific interest over the years [20,21,22,23,24,25]. The most characteristic feature of PNIPAM is that although it is water soluble at room temperature, upon heating the solution above 32 °C, which is its specific low critical solution temperature (LCST), a reversible coil-to-globule phase transition occurs [20]. This transition from an extended soluble hydrated state to a shrunken insoluble dehydrated state is directly correlated to the formation of intermolecular hydrogen bonds and the increase in hydrophobic interactions that lead to extensive polymer chain aggregation. The fact that the LCST of PNIPAM happens to be close to body temperature renders it an ideal candidate for biorelevant applications like gene transfer.

Herein, we investigate the electrostatic interaction between a chitosan grafted with PNIPAM side chains, denoted as Chit-*g*-PNIPAM, and two DNA macromolecules of different lengths approximately corresponding to 50 or 2000 bp or, in other words, a short nucleic acid versus a long one. The aim of our study was the construction of stable polyplexes that can demonstrate the potential use of Chit-*g*-PNIPAM in gene delivery systems and the investigation of their physicochemical properties, especially in regard to the effect of the DNA length. The binding between the chitosan graft copolymer and the DNA macromolecules is readily enabled by the electrostatic interaction of the protonated amino groups of the chitosan backbone with the oppositely charged phosphate groups of the DNA chains. At the same time, the PNIPAM side chains are expected to impart thermal responsiveness to the formed polyplexes that can affect the interaction between the DNA vectors and cell membranes. The physicochemical properties in regard to the mass, size, size distribution, and effective charge of the resulting polyplexes formed at different amino to phosphate group (N/P) mixing ratios were investigated by dynamic and electrophoretic light scattering (DLS and ELS). In parallel, scanning-transmission electron microscopy (STEM) was used in order to elucidate their morphology. Due to the thermoresponsive character of the PNIPAM chains, the response of the formed Chit-*g*-PNIPAM+DNA polyplexes to the variation in temperature was investigated by means of DLS measurements. Furthermore, their stability against the increase in the ionic strength and/or upon interaction with biological media, such as fetal bovine serum (FBS), was also examined by monitoring changes in their mass and size through DLS. Finally, fluorescence spectroscopy measurements using ethidium bromide as a probe were employed in order to evaluate the DNA-binding affinity of the chitosan graft copolymer, while the secondary structure of the incorporated DNA chains was probed by infrared spectroscopy.

## 2. Materials and Methods

### 2.1. Materials

The water-soluble chitosan (containing lactate and phosphate counterions) used in this study has an average molecular weight of *M*_W_ = 162,000 g/mol, a degree of deacetylation (DD) of 88.2%, was purchased from Shandong AK Biotech Co LTD (Jinan, China), and was used as received. The corresponding PNIPAM homopolymer used for the grafting of chitosan was synthesized in house as described in detail elsewhere [26] and has an average molecular weight of *M*_W_ = 3200 g/mol, as shown by size exclusion chromatography (SEC) measurements. All the necessary reagents like potassium persulfate (KPS, 99%), ethidium bromide (EtBr), sterile-filtered fetal bovine serum (FBS), and phosphate buffer saline (PBS) tablets were purchased from Sigma-Aldrich (Sigma Chemical Co.; St. Louis, MO, USA). All aqueous solutions were prepared in water for injection (WFI), so as to have a stable pH around 6.5. Deoxyribonucleic acid from herring sperm with a length < 50 bp (D3159) and deoxyribonucleic acid sodium salt from salmon testes with an approximate length of 2000 bp (D1626) were also received from Sigma-Aldrich (Sigma Chemical Co.; St. Louis, MO, USA).

### 2.2. Synthesis of the Chit-g-PNIPAM Copolymer

The Chit-*g*-PNIPAM copolymer used in this study was synthesized following the “grafting to” approach, which entails the anchoring (via a radical coupling reaction) of PNIPAM side chains onto the polysaccharide backbone and has been described in detail in a previous publication of our group [26]. In brief, first the PNIPAM homopolymer was synthesized by reversible addition–fragmentation chain transfer polymerization (RAFT) of *N*-isopropylacrylamide (NIPAM), followed by the free-radical grafting of chitosan with the active PNIPAM homopolymer (a macromolecular chain transfer agent) in aqueous solution using potassium persulfate as the supplementary radical-producing agent. The excess/unbonded PNIPAM was removed by dialysis in water and the final product was obtained in solid form after removal of the solvent by freeze-drying. The structure of the resulting Chit-*g*-PNIPAM copolymer was confirmed through nuclear magnetic resonance (NMR) spectroscopy and the weight percentage of PNIPAM in the graft copolymer was found to be about 19.9%. Based on the *M*_W_ and DD of the precursor chitosan sample (i.e., 162,000 g/mol and 88.2%, respectively) we calculate about 975 monomeric units (860 deacetylated and 114 acetylated monomeric units) for the polysaccharide chain, while the corresponding *M*_W_ for the synthesized PNIPAM homopolymer approximately equals 25 monomeric units. According to the weight percentage of PNIPAM (i.e., 19.9%) there are 356 monomeric units of NIPAM per Chit-*g*-PNIPAM chain and since each PNIPAM chain has 25 monomer units, there are about 14 PNIPAM chains per grafted chitosan backbone. Therefore, the molecular weight of the Chit-*g*-PNIPAM copolymer is approximatively 206,800 g/mol.

### 2.3. Preparation of Chit-g-PNIPAM and DNA Polyplexes

In order to investigate the electrostatic interaction between Chit-*g*-PNIPAM and the two DNA samples of different lengths, a series of polyplex dispersions at varying mixing ratios between the two macromolecular components were prepared. Specifically, as a first step, initial stock solutions of the graft copolymer at a concentration of 0.1 mg/mL in 0.5% *v*/*v* acetic acid (thus facilitating the full protonation of the chitosan amino groups) and the two DNA samples at 0.14 mg/mL concentration in WFI were prepared and left to equilibrate overnight. Next, appropriate volumes of each DNA solution, that is, 0.25, 0.5, 1, and 2 mL, were added to 1 mL of the Chit-*g*-PNIPAM solution under stirring. In some cases, the formation of large polyplexes or aggregates could be macroscopically observed during this stage by the increase in the turbidity of the dispersions. As a final step all polyplex dispersions were diluted with WFI to the same final volume of 5 mL, so as to have the same concentration of Chit-*g*-PNIPAM throughout the series of samples. It should be noted that the pH of the polyplex dispersions is equal to that of WFI (due to the final dilution), which measures at about 6.5. The concentrations and mixing volumes of the initial stock solutions were specifically chosen so that the estimated molar ratio of amino to phosphate groups, N/P, of the polyplexes is equal to 0.5, 1, 2, and 4. After overnight equilibration the polyplex dispersion at N/P = 0.5 of the Chit-*g*-PNIPAM+DNA50 and the corresponding one at N/P = 1 of the Chit-*g*-PNIPAM+DNA2000 system exhibited some precipitation and the corresponding measurements were performed on the supernatant. The characteristics of the polyplex samples related to the final concentrations and N/P mixing ratios of the two components are summarized in Table 1.

The response of the formed polyplexes against the increase in the ionic strength was also examined by titrating 1 mL of representative stable polyplex dispersions at N/P = 4 of both Chit-*g*-PNIPAM+DNA50/2000 systems with appropriate aliquots of a 1 M NaCl solution, covering a range of ionic strength up to 0.5 M. The titration was monitored by means of DLS measurements and was performed directly in the instrument sample cell. After each addition step, the system was allowed to equilibrate for 10 min before measurement. In parallel, the behavior of the same representative stable polyplexes (formed at N/P = 4) of both systems upon interaction with biological fluids such as FBS was investigated. Stock solutions of FBS in PBS at an FBS:PBS volume ratio of 1:9 or 1:1 (that is, 10 or 50% *v*/*v*) were prepared and filtered through 0.45 μm hydrophilic PVDF syringe filters (ALWSCI Group, Hangzhou, China). Afterwards, equal volume ratio mixtures (i.e., 1:1) of the two FBS solutions of different concentrations with each of the stable polyplex dispersions of the two Chit-*g*-PNIPAM+DNA50/2000 systems were prepared and left to equilibrate for 30 min. Finally, DLS measurements of the mixtures were performed both at 25 °C (ambient conditions) and after incubation at 37 °C (body temperature) for 30 min.

### 2.4. Dynamic and Electrophoretic Light Scattering

DLS measurements were performed utilizing the Litesizer 500 device from Anton Paar (Graz, Austria) equipped with a 40 mW single-frequency laser diode operating at a 658 nm wavelength and analyzed with the Kalliope software (version 3.2.4). Measurements were performed at a scattering angle of 90°, either at ambient conditions or as a function of temperature, and information on the scattered light intensity, particle size, size distribution, and polydispersity index of the polyplexes was obtained. For the temperature-dependent measurements, the temperature was raised from 25 to 45 °C with 5 °C steps followed by cooling of the samples back to 25 °C, with each measurement starting after a 10 min equilibration period. All obtained values are derived from mean measurements usually averaging five consecutive runs of ten seconds. In a similar manner, ELS measurements were conducted on the same instrument at a 175° scattering angle and at room temperature, providing the zeta potential of the polyplexes. It should be noted that for the measured scattered intensity values, there is a standard deviation of approximately 1–2%, while for the calculated hydrodynamic radius and zeta potential values, the corresponding standard deviation is about 5%.

### 2.5. Scanning-Transmission Electron Microscopy

The morphology of the formed polyplexes was evidenced using a Verios G4 UC (Thermo Fisher Scientific, Waltham, MA, USA) scanning electron microscope in high-vacuum mode using the retractable specific detector STEM 3+ working at 20 kV. The polyplexes were deposited on 300 mesh copper grids coated with lacey carbon film.

### 2.6. Fluorescence Spectroscopy

The DNA-binding ability of the Chit-*g*-PNIPAM copolymer at various N/P ratios was evaluated by means of fluorescence spectroscopy measurements utilizing ethidium bromide as a probe. In more detail, stock solutions of both DNA samples at 14 μg/mL were prepared in WFI, followed by addition of ethidium bromide at a molar ratio [EtBr] = [P]/4, where [P] is the molar concentration of DNA phosphate groups. The DNA solutions pretreated with EtBr were left to equilibrate overnight. Subsequently, the DNA aqueous solutions containing the EtBr were titrated with appropriate volumes of a stock solution of the Chit-*g*-PNIPAM copolymer at 0.1 mg/mL concentration in 0.5% wt. acetic acid, covering a range of N/P ratios from 0 to 6. After each titration step, the dispersions were equilibrated for 15 min at 25 °C before measurements. The spectra were recorded with a double-grating excitation and a single-grating emission spectrofluorometer (Fluorolog-3, model FL3-21, Jobin Yvon-Spex, Horiba Ltd., Kyoto, Japan) at room temperature. The excitation wavelength used was *λ* = 535 nm and the emission spectra were recorded in the region 555–800 nm, with an increment of 1 nm, using an integration time of 0.5 s, and slit openings of 4 nm for both the excitation and the emitted beam.

### 2.7. Fourier Transform Infrared Spectroscopy

The measurements were performed at room temperature in the spectral range of 5000–550 cm^−1^ using an Equinox 55 mid-infrared FTIR spectrometer by Bruker (Bruker GmbH, Ettlingen, Germany). The spectrometer is equipped with a single-bounce attenuated total reflectance (ATR) diamond accessory (DuraSamplIR II by SensIR Technologies, Danbury, CT, USA). Background spectra were obtained by recording the pristine and desiccated ATR diamond crystal surface in ambient air. The measurements of the polyplex dispersions were performed in the dry state, after creating a thin film of the dispersion’s components directly on the ATR diamond crystal by solvent evaporation under N_2_ flow. For each sample, the final spectrum is the average of three 100-scan measurements at 2 cm^−1^ resolution. Appropriate corrections have been made on the final spectra to eliminate contribution from H_2_O vapor bands. Moreover, the spectral contribution of the pure Chit-*g*-PNIPAM copolymer was suitably subtracted from the final spectra.

## 3. Results and Discussion

### 3.1. Physicochemical Properties of the Chit-g-PNIPAM+DNA Polyplexes

**Figure 1:**
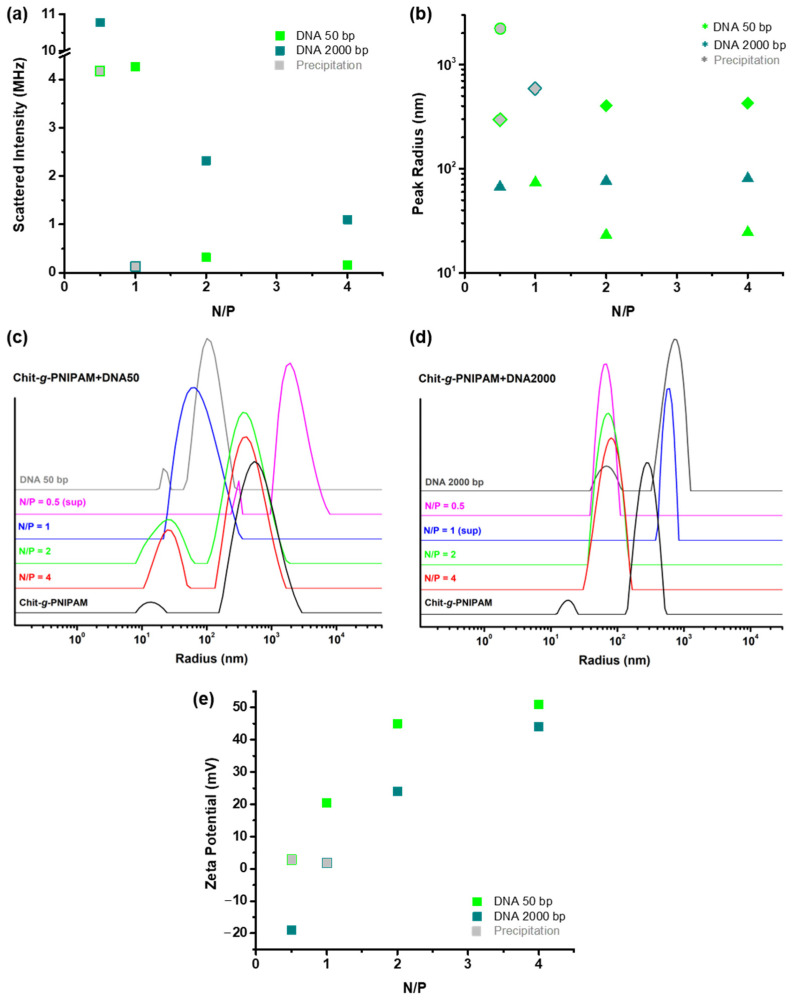
Overall DLS and ELS results in regard to (**a**) the scattered intensity, (**b**) the hydrodynamic radius derived from the peaks (various symbols denote different peaks according to size) of the corresponding (**c**,**d**) size distribution functions (SDFs), and (**e**) the zeta potential values, for the polyplexes of the two Chit-*g*-PNIPAM+DNA50/2000 systems, as a function of the N/P ratio. Note that in (**a**,**b**,**e**) values derived from measurements of the supernatant of precipitated polyplex dispersions are denoted by grey filled symbols, while in (**c**,**d**) the corresponding SDFs are marked as: (sup).

A series of DLS and ELS measurements were conducted in order to examine the physicochemical properties of the Chit-*g*-PNIPAM+DNA polyplexes formed at different N/P ratios for both DNA samples (50 and 2000 bp) under study. The obtained results regarding the scattered intensity, the values of the hydrodynamic radius (*R*_h_) of the various scattering populations discerned, evidenced by the different peaks of the corresponding size distribution functions (SDFs), with the SDFs for the pure components are also included for comparison, along with the zeta potential of the polyplexes are shown in Figure 1, as a function of the N/P ratio. As mentioned in the description of the preparation of the polyplexes (see Section 2.3), the dispersion at N/P = 0.5 for the short DNA series (50 bp) and the one at N/P = 1 for the long one (2000 bp) exhibited precipitation after equilibration, which of course is a direct indication of extended aggregation. For these samples the measurements were conducted on the supernatant and the corresponding obtained data are denoted appropriately.

As seen in Figure 1a, the mass (which is directly proportional to the scattered intensity) of the polyplexes formed between the Chit-*g*-PNIPAM graft copolymer and either the short or the long DNA exhibits its highest values for N/P ratios below 1 (i.e., estimated charge neutralization point). Please note that for the precipitated dispersions the measured intensity values correspond to only a small fraction of the total mass (since most of the polyplexes/aggregates have precipitated), otherwise they would have been much higher. Therefore, it can be deduced with relative certainty that, for N/P ≤ 1, the interaction between the two components is extremely strong or, in other words, the excess of DNA related to the available amino groups of Chit-*g*-PNIPAM leads to the formation of large/massive polyplexes or aggregates. These formations are most probably colloidally unstable due to charge neutralization, along with their increased mass, thus undergoing secondary aggregation and eventually precipitating. As far as the comparison between the two DNAs is concerned, it seems that at N/P = 1 the polyplexes formed with the short DNA do not reach the stoichiometry (no precipitation observed) of those formed with the long one which already precipitated at this ratio. Apparently, the long DNA enables to a greater extent the secondary aggregation of the polyplexes, probably acting as a bridging agent between them. At the same time, when the DNA2000 sample is in excess at N/P = 0.5, it looks like it provides additional charges and thus the formed polyplexes/aggregates are still stable although very massive and/or compact, as evidenced by the extremely high scattered intensity. By the same reasoning, the fact that the short DNA forms stable polyplexes close to the nominal charge neutralization point (N/P = 1) could be an indication of its greater ability to be incorporated and efficiently accommodated into the formed polyplexes, due to its smaller size. Nevertheless, as the content of the DNA50 sample increases further (N/P = 0.5), extensive aggregation (leading to precipitation) is also induced, likely as a result of the large-scale charge neutralization. When the Chit-*g*-PNIPAM copolymer is in excess and thus there is a surplus of positive charges, that is, for N/P > 1, the resulting polyplexes are less massive/dense and colloidally stable. This is directly correlated to the solubility of the graft copolymer (due also to the contribution of soluble non-interacting PNIPAM chains) and the fact that the formed polyplexes comprise an increased number of Chit-*g*-PNIPAM chains, especially in comparison to the ones formed at lower N/P ratios. Lastly, it is worth mentioning that for the same N/P values the polyplexes formed with the long DNA have a higher mass or are more compact than the ones with the short DNA, possibly indicating a higher degree of interaction with the graft copolymer.

Regarding the size of the formed polyplexes, the obtained SDFs shown in Figure 1c,d reveal the presence of various peaks providing valuable insights. First of all, the pure Chit-*g*-PNIPAM solution exhibits two peaks indicating two scattering populations in solution, with corresponding *R*_h_ values in the range of 10 to 20 nm for the small one and about 600 or 300 nm for the large one. This difference in the size of the second peak between the two SDFs is attributed to the fact that they correspond to different prepared stock Chit-*g*-PNIPAM solutions. Still, the presence of the second large peak indicates that the graft copolymer exhibits some degree of self-assembly forming multi-chain aggregates, most probably due to hydrophobic interactions stemming from the chitosan backbone or intra-polyelectrolyte interactions of the copolymer. Hence, most probably both single Chit-*g*-PNIPAM copolymer chains and multi-chain aggregates coexist in the solution. The same stands for the two DNA samples as well, since they also exhibit two peaks, with *R*_h_ values about 20 and 100 nm for the short DNA or about 70 and 700 nm for the long one, in direct proportion to the respective lengths of the two nucleic acids. As far as the polyplexes of the Chit-*g*-PNIPAM+DNA50 system are concerned, at high N/P values (i.e., 2 and 4) the SDFs are similar to that of the pure copolymer, or in other words the sizes of the two populations of polyplexes are dictated by the corresponding populations of the graft copolymer which is in excess. More specifically, the smaller population has an *R*_h_ of about 20 nm (Figure 1b), somewhat larger both in size and overall intensity compared to that of the pure Chit-*g*-PNIPAM, which is additional confirmation of the formation of polyplexes. Note that this population most likely indicates polyplexes formed between the original single copolymer chains and DNA molecules. In parallel, the second peak denotes an *R*_h_ of about 400 nm, which is somewhat smaller than the size of the multi-chain aggregates of the copolymer. So, it seems that there is some shrinking of the Chit-*g*-PNIPAM aggregates upon binding of the DNA50 molecules, probably due to the reduction of electrostatic repulsions caused by charge neuralization and screening. This effect is even more pronounced at N/P = 1, where only one peak with an intermediate size around 75 nm is observed, indicating the formation of compact/dense polyplexes (also evidenced by the increased scattered intensity), possibly incorporating a large number of Chit-*g*-PNIPAM copolymer chains and short DNA molecules. For the precipitated dispersion at N/P = 0.5 an additional population of very large aggregates (*R*_h_ ≈ 2 μm) can still be detected in the supernatant, denoting the occurring extended secondary aggregation. Quite interestingly, for the Chit-*g*-PNIPAM+DNA2000 system the SDFs show only one peak/population, with a rather similar size of about 75 nm for all three stable dispersions. This signifies the formation of compact and/or dense polyplexes regardless of the aggregation state and content of the graft copolymer, which is obviously a direct consequence of the length of the specific DNA sample. This means that the long DNA molecules cannot be incorporated in the pre-existing multi-chain aggregates of the Chit-*g*-PNIPAM copolymer without changing their structure much (as in the case of the short DNA sample). Again, in the case of the dispersion showing precipitation (N/P = 1) a relatively larger population (*R*_h_ ≈ 600 nm) compared to the rest can be discerned, indicative of the secondary aggregation.

The information about the effective charge of the formed polyplexes, derived from the acquired zeta potential values shown in Figure 1e, are in line with the assumptions made so far. Starting at the highest N/P value, both Chit-*g*-PNIPAM+DNA50/2000 systems exhibit relatively strong positive values, close to the corresponding one measured for the pure copolymer, which is about +50 mV. This observation confirms that when the graft Chit-*g*-PNIPAM copolymer is in excess, the overall properties and conformation of the formed polyplexes are mostly dictated by the copolymer’s intrinsic conformational/aggregation state and characteristics. As the N/P ratio decreases (by increasing the DNA content), a gradual decrease in the zeta potential of the polyplexes can be observed, reaching values close to zero for the short DNA sample, or even negative values (i.e., charge reversal) in the case of the long one. It is thus confirmed that the polyplexes formed at N/P ratios close to 1 are characterized by reduced overall charge, as a result of the charge neutralization that takes place upon the electrostatic interaction of the two components. Of course, a reduction of the overall charge of the polyplexes (or in other words zeta potential values close to zero) also entails colloidal instability, which leads to the observed precipitation. Apparently, the presence of PNIPAM soluble chains cannot prevent precipitation of the formed polyplexes. One final point worth noting is that for the long DNA, the polyplexes formed at N/P = 0.5 show negative zeta potential as a result of the excess of DNA, which also imparts additional stability to the system. It seems that the increased conformational constraints of the longer DNA chains prevent them from being fully incorporated into the formed polyplexes, in contrast to the shorter DNA macromolecules.

**Figure 2:**
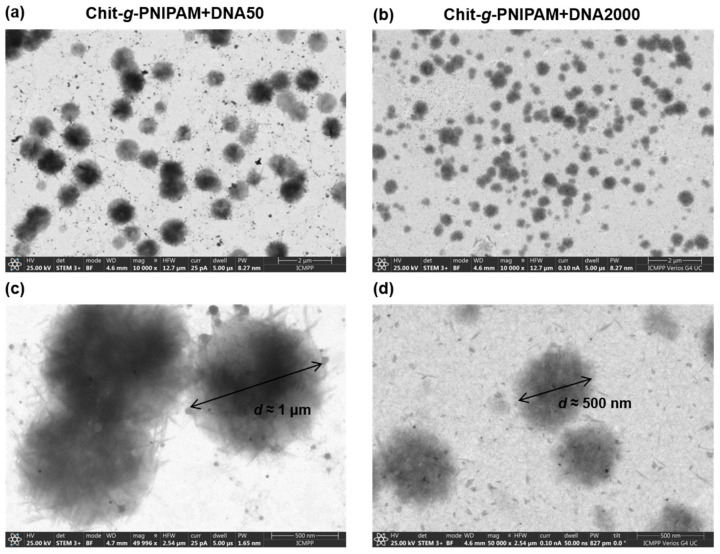
STEM images for the stable polyplexes formed at N/P = 4 of both (**a**,**c**) Chit-*g*-PNIPAM+DNA50 and (**b**,**d**) Chit-*g*-PNIPAM+DNA2000 systems, at (**a**,**b**) low and (**c**,**d**) high magnification.

In order to gain further insight regarding the morphology of the polyplexes, STEM imaging was performed on the polyplexes formed at N/P = 4 for both Chit-*g*-PNIPAM+DNA50/2000 systems, as shown in Figure 2. In both cases spherical homogeneous nanostructures of various sizes and rather loose, urchin-like structures are observed. Obviously, the density of the polyplexes decreases close to their periphery and rather straight or slightly curved bundles of complexed copolymer/DNA chains are visible in the outer parts of the formed nanostructures (especially in the DNA50 case). The latter characteristic may be a result of the semirigid nature of DNA and chitosan backbone and hint towards a ladder-like coassembly of the primary DNA/graft copolymer complexes. However, DNA2000 may be more well condensed in the primary complexes due to its longer length that gives it high conformational adaptability upon complexation. Notably, for the Chit-*g*-PNIPAM+DNA50 system there is a better distinction between populations of different sizes (i.e., small vs. large particles), while quite the opposite stands for the Chit-*g*-PNIPAM+DNA2000 system, where a broader distribution of sizes is observed. Nevertheless, the overall size of the polyplexes formed between the graft Chit-*g*-PNIPAM copolymer and the short DNA sample is bigger than that of the ones formed with the long DNA, in accordance with the corresponding DLS results. As seen more clearly in the higher-magnification images (Figure 2c,d), the large polyplexes corresponding to the DNA50 or DNA2000 sample have a diameter of about 1 μm or 500 nm, respectively. In general, the size derived from the STEM images is somewhat larger than the hydrodynamic size measured by means of DLS, because nanostructures of loose morphology like the ones under study are expected to adopt a more flattened conformation upon surface deposition and after solvent evaporation. Still, there seems to be a greater inconsistency between the two methods for the Chit-*g*-PNIPAM+DNA2000 system, with the STEM size of the polyplexes (radius about 250 nm) being significantly larger than the corresponding *R*_h_ value (~75 nm). One possible explanation for this is that since the distribution of sizes is broader in this case, the DLS technique is not able to fully distinguish between different populations and thus underestimates to some extent the size of the larger polyplexes.

### 3.2. Temperature Response of the Chit-g-PNIPAM+DNA Polyplexes

**Figure 3:**
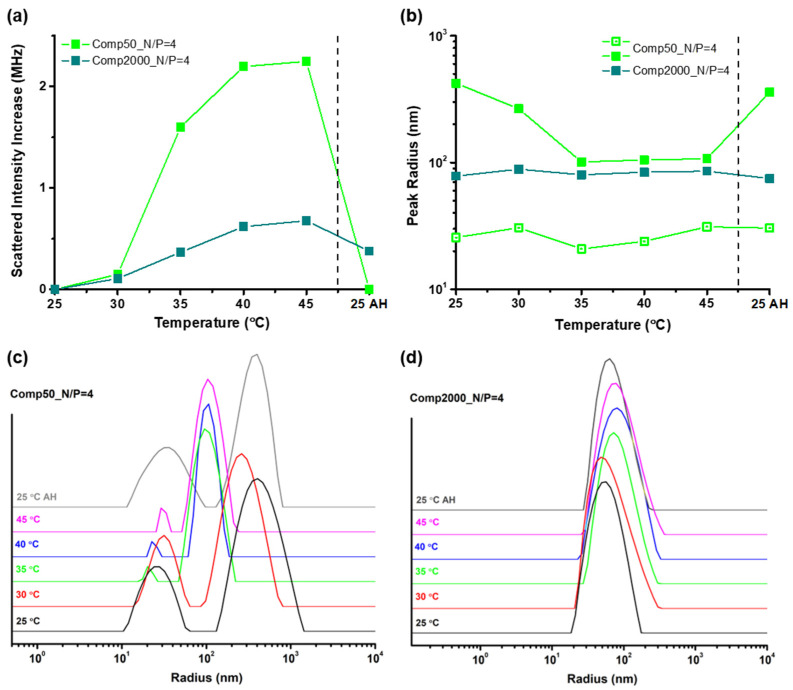
The influence of temperature on (**a**) the scattered intensity and (**b**) the hydrodynamic radius derived from the peaks (open and filled green symbols denote different peaks according to size) of the corresponding (**c**,**d**) SDFs, for the stable polyplexes formed at N/P = 4 of both (**c**) Chit-*g*-PNIPAM+DNA50 and (**d**) Chit-*g*-PNIPAM+DNA2000 systems. The after heating (AH) values are separated by a dashed line.

As anticipated, the presence of the PNIPAM side chains imparts a thermoresponsive character to the Chit-*g*-PNIPAM graft copolymer [26], which can be exploited in potential bioapplications like gene delivery. More specifically, the temperature-induced increase in hydrophobic interactions (due to the phase transition of PNIPAM chains) could potentially facilitate the interaction with cell membranes and thus enhance cellular uptake. Therefore, the investigation of the thermal response of the already formed Chit-*g*-PNIPAM+DNA50/2000 polyplexes is of particular interest. For this reason, DLS measurements of representative polyplex stable dispersions from both systems at a range of temperatures from 25 to 45 °C, i.e., within the biologically relevant temperature range, were performed. Figure 3 presents the obtained results in regard to the increase in the scattered intensity (i.e., the initial values have been shifted to zero for clarity), and the *R*_h_ values of the different peaks discerned in the corresponding SDFs, as a function of temperature, for the stable polyplexes formed at N/P = 4 of both Chit-*g*-PNIPAM+DNA50/2000 systems. Similar measurements were also performed for the non-precipitated dispersions at the lowest N/P values of each system (i.e., N/P = 1 or 0.5 for the DNA50 or DNA2000 samples, respectively), as well as the pure Chit-*g*-PNIPAM copolymer solution for comparison, and the results can be found in the Supplementary Materials (see Figures S1 and S2). Note that in all cases after the gradual increase in temperature up to 45 °C, the sample was cooled back to 25 °C and remeasured in order to examine the reversibility of any occurring changes. These measurements are marked as AH, which stands for “after heating”, and the corresponding values in Figure 3a,b are separated by a dashed line.

A significant increase in the scattered intensity is observed above 35 °C for both systems, as seen in Figure 3a, with the change being even more pronounced in the case of the polyplexes formed with the short DNA sample. This behavior is also evidenced by the pure Chit-*g*-PNIPAM graft copolymer (see Figure S2), which is attributed to the increase in the hydrophobic interactions and is considered a characteristic trait of the thermoresponsive nature of PNIPAM. At the same time, the size of the DNA2000-based polyplexes seems to be rather unaffected by the increase in temperature, with only a slight broadening of the *R*_h_ peak being discerned in the corresponding SDFs (Figure 3d). On the contrary, for the DNA50 system a considerable decrease in the size of the larger population is witnessed (Figure 3c), while for the size of the smaller population there is a much slighter decrease mostly observed at 35 °C. Note that the Chit-*g*-PNIPAM copolymer demonstrates an analogous decrease in size (Figure S2c). Taking all these observations into account, it seems that the large population of polyplexes formed with the short DNA sample have a rather loose conformation/structure, resembling to a large degree that of the multi-chain aggregates of the graft copolymer (see also the corresponding images in Figure 2a,c). Thus, upon heating they transition to more compact/collapsed configurations (also evidenced by the drastic increase in scattered intensity) due to the increase in the hydrophobic interactions between the PNIPAM side chains (either with one another or with the chitosan backbone). This effect appears less intense in the case of the smaller in size population, probably meaning that they are characterized by a more compact initial conformation that does not really allow for further shrinking upon heating. This also applies for the Chit-*g*-PNIPAM+DNA2000 polyplexes whose size exhibits no significant changes, again indicating an increased level of initial compactness. However, it is possible that a small degree of secondary aggregation between the polyplexes occurs, as indicated by the broadening of the *R*_h_ peak and the not so drastic, yet observable, increase in the scattered intensity, owing to the increase in the hydrophobic interactions as temperature rises. Likewise, secondary aggregation upon heating is possibly taking place in the case of the polyplexes formed at N/P = 1 of the Chit-*g*-PNIPAM+DNA50 system, according to the observed increase in both the scattered intensity (which is particularly intense) and the corresponding size (see Figure S1). On the contrary, the polyplexes corresponding to N/P = 0.5 for the DNA2000 sample are practically uninfluenced by the increase in temperature (see Figure S1), most probably because of the excess of DNA that imparts additional colloidal stability to the system. Overall, the thermal response of the formed polyplexes is closely linked to their conformation/structure, which in turn is a direct consequence of the N/P ratio and the DNA length. Nevertheless, the observed changes are essentially fully reversible in all cases, showcasing the adaptability and resilience of the formed polyplexes.

### 3.3. Stability of the Chit-g-PNIPAM+DNA Polyplexes

**Figure 4:**
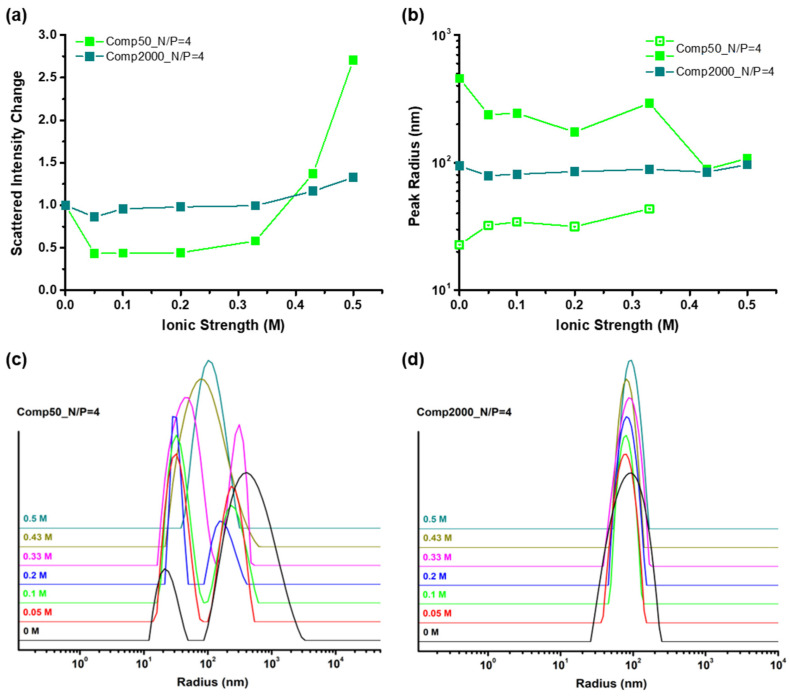
The influence of ionic strength on (**a**) the scattered intensity and (**b**) the hydrodynamic radius derived from the peaks (open and filled green symbols denote different peaks according to size) of the corresponding (**c**,**d**) SDFs, for the stable polyplexes formed at N/P = 4 of both (**c**) Chit-*g*-PNIPAM+DNA50 and (**d**) Chit-*g*-PNIPAM+DNA2000 systems.

Another important factor for the successful application of potential gene carrier systems, like the ones under consideration, is their stability in various environments. On these grounds, the effect of the increase in the ionic strength on the stable polyplexes of both systems was examined by means of appropriate DLS measurements and the results are shown in Figure 4. Namely, the change in the scattered intensity (i.e., the initial values have been normalized to one for clarity) and the *R*_h_ values of the different peaks from the corresponding SDFs, for the polyplex dispersions at N/P = 4 of both Chit-*g*-PNIPAM+DNA50/2000 systems, are plotted against the ionic strength. The same experimental protocol was also applied on the pure Chit-*g*-PNIPAM copolymer solution for comparison and the obtained results are presented in a similar manner in Figure S3.

As seen in Figure 4a, the initial addition of salt (0.05 M) in the dispersion of the polyplexes prepared with the short DNA causes a noticeable drop in the scattered intensity, as well as the size of the larger population discerned from the relative SDFs (note that there is also an evident change in the relative intensities of the two peaks). A similar transition is observed for the pure Chit-*g*-PNIPAM copolymer as well (see Figure S3), strengthening the assumption that at low ionic strength values the solubility of the graft copolymer increases due to the reduction in the hydrophobic interactions, thus leading to some degree of dissociation of the initial multi-chain copolymer aggregates. Most probably, this is also the case for the corresponding polyplexes formed between the copolymer aggregates and the DNA molecules. A further increase in the ionic strength up to 0.33 M appears to have no significant effect either on the conformation or the size of the formed polyplexes, which is a good indication of their stability. Even so, when reaching high ionic strength (above 0.43 M) an abrupt increase in the scattered intensity is observed, accompanied by a transition from two to one peak in the corresponding SDFs. It seems that at high salt content the Chit-*g*-PNIPAM+DNA50 polyplexes collapse to more compact/dense structures due to the increase in the hydrophobic interactions associated with the worsening of the solubility of the copolymer (see Figure S3) and the extended charge screening. Moving on to the Chit-*g*-PNIPAM+DNA2000 system, analogous transitions of the scattered intensity and the size of the polyplexes are taking place (i.e., initial drop followed by small fluctuations and eventual increase) but to a much smaller variation range. This behavior probably stems from the already more compact initial conformation of these polyplexes that apparently prevents them from adopting more collapsed structures. On the whole, although some conformational rearrangements are inflicted, the polyplexes of both systems exhibit remarkable stability, especially at intermediate ionic strength values that are relevant to biological applications (i.e., about 0.15 M for blood plasma or cell cytoplasm).

**Figure 5:**
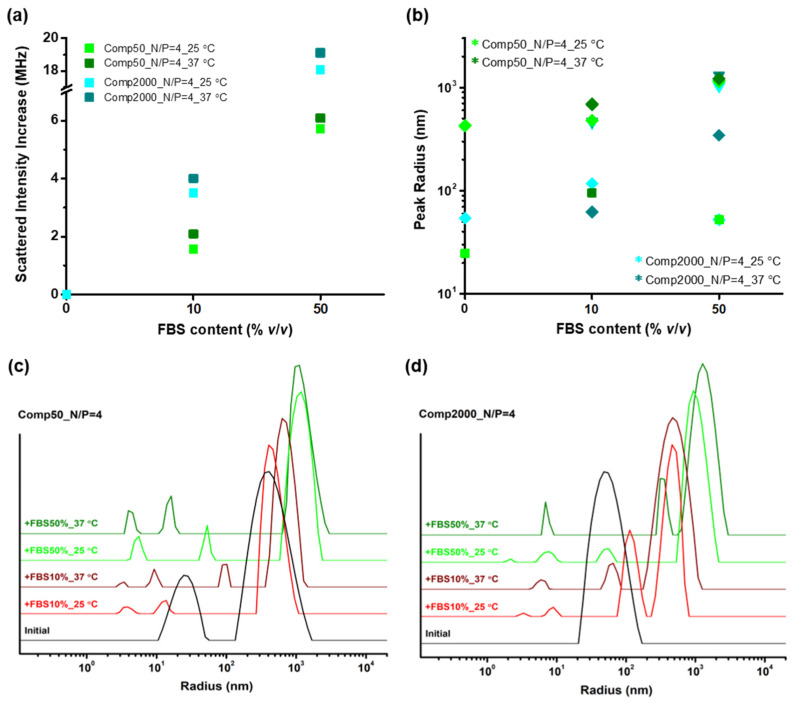
The influence of interaction with FBS on (**a**) the scattered intensity and (**b**) the hydrodynamic radius derived from the peaks (various symbols denote different peaks according to size, excluding the ones attributed to FBS) of the corresponding (**c**,**d**) SDFs, for the stable polyplexes formed at N/P = 4 of both (**c**) Chit-*g*-PNIPAM+DNA50 and (**d**) Chit-*g*-PNIPAM+DNA2000 systems and the two FBS solutions of different contents (10 and 50% *v*/*v*).

Apart from the stability against the ionic strength, it is equally important to test the behavior of the polyplexes when interacting with biological fluids, as a means of simulating their response upon introduction into the human body. For this purpose, parts from the stable polyplex dispersions of both systems were mixed (in equal volumes) with FBS solutions of two different concentrations, i.e., 10 and 50% *v*/*v* in PBS, and the final equilibrated mixtures were measured by DLS first at room temperature (25 °C) and subsequently at body temperature (37 °C) after incubation for 30 min. Figure 5 presents the obtained results regarding the increase in the scattered intensity (i.e., the initial values have been shifted to zero for clarity), and the *R*_h_ values of the different peaks from the corresponding SDFs (which are also shown in Table S1 for better clarity), for the polyplexes at N/P = 4 of both Chit-*g*-PNIPAM+DNA50/2000 systems, after mixing with FBS solutions of different contents (10 and 50% *v*/*v*). Note that values at zero FBS content denote the initial state of the dispersions. Moreover, the small peaks with sizes below 20 nm seen in the SDFs are attributed to FBS (see Figure S5) and have been excluded from Figure 5b for clarity reasons. The interaction of the pure Chit-*g*-PNIPAM copolymer with FBS was also examined in a similar manner and the corresponding results are shown in Figure S4, while Figure S5 displays relevant DLS results for the neat FBS solutions.

The DLS results reveal an increase mainly in the scattered intensity and to a lesser extent the size of the stable polyplexes of the DNA50 sample after mixing with the FBS solutions, with the differences being more pronounced for the high FBS content. Even after incubation at 37 °C small changes are observed. Therefore, it can be assumed that although the polyplexes interact with the components of the FBS solution (mostly proteins and enzymes), a fact that cannot be disputed since there is a considerable increase in the scattered intensity, their size does not increase much most probably due to their initial loose structure that allows for appropriate conformational rearrangements. Of course, at higher FBS content each polyplex binds more proteins/enzymes, resulting in a noticeable increase in both their mass and size. Still, no extended secondary aggregation that could lead to precipitation is observed in any case, or in other words, the resulting mixed particles are essentially stable. To some extent this should be attributed to the presence of the soluble not interacting with PNIPAM grafted chains of the copolymer component. Moreover, they seem to have a rather compact/dense configuration that is mostly unaffected by the increase in temperature and the consequent increase in hydrophobic interactions. Roughly the same narrative stands for the polyplexes of the Chit-*g*-PNIPAM+DNA2000 system as well, the main difference in this case being that these polyplexes are already characterized by an initial compact conformation, thus upon binding of the FBS components a significant increase in their mass and size occurs, especially at high FBS content, where the possibility of secondary aggregation is rather likely. Nevertheless, they also exhibit remarkable stability against biological fluids (no precipitation or phase separation observed), even after incubation at body temperature. Surely, the behavior of the polyplexes originates to some degree from the precursor Chit-*g*-PNIPAM copolymer, which demonstrates similar properties (see Supplementary Materials).

### 3.4. DNA-Binding Affinity of the Chit-g-PNIPAM Copolymer

**Figure 6:**
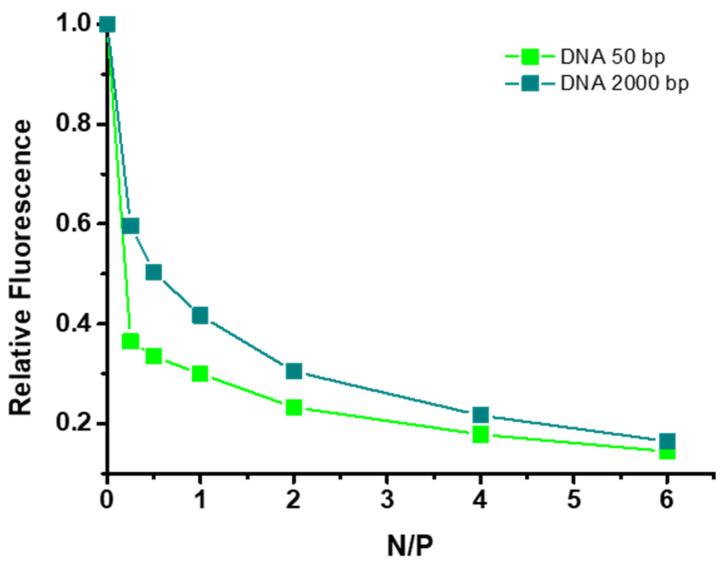
EtBr fluorescence quenching in the polyplexes formed between the Chit-*g*-PNIPAM copolymer and the two DNA samples.

A well-established method for the investigation of the DNA-binding affinity of various cationic polymers is based on EtBr fluorescence-quenching assays [27,28,29,30,31]. Ethidium bromide is a strong fluorescent cationic dye that can be easily intercalated into the base pairs of DNA molecules. When DNA chains interact with cationic polymers, EtBr is displaced from the DNA helix, causing a quenching of the fluorescence intensity. In this manner, both DNA samples used in this study (i.e., 50 and 2000 bp) were labeled with EtBr and subsequently titrated with the Chit-*g*-PNIPAM copolymer in a range of N/P ratios from 0 (initial labeled DNA solution) up to 6. The titration was followed spectroscopically and the relative fluorescence intensities for both DNA samples at the different N/P values are shown in Figure 6, while the corresponding acquired emission spectra are presented in Figure S6.

It is quite obvious that in both cases a significant quenching of the fluorescence of the EtBr occurs upon binding with the Chit-*g*-PNIPAM copolymer, which is a direct proof of the formation of polyplexes between the two macromolecular components. Moreover, this decrease in intensity is more abrupt/steep for the short DNA sample, since a significant drop in the fluorescence (more than 60% of the initial value) takes place even at the lowest N/P ratio. Therefore, one can infer that the Chit-*g*-PNIPAM copolymer shows greater binding affinity towards the short nucleic acid or, in other words, that it is easier for the DNA50 chains to interact with the graft copolymer and be incorporated into the formed polyplexes. This effect is of course a direct consequence of the length of the DNA molecule and its conformational properties (a more rod-like conformation) and is thus more prominent at lower N/P ratios, where the DNA is in excess, and a large number of DNA molecules interact with each Chit-*g*-PNIPAM copolymer chain. Another point worth making is that at the end of the titration (N/P = 6) both systems reach practically the same final degree of quenching, which demonstrates the capacity of the graft copolymer to fully incorporate both DNA molecules (independently of their length) when it is in excess.

### 3.5. DNA Structure Within the Chit-g-PNIPAM+DNA Polyplexes

**Figure 7:**
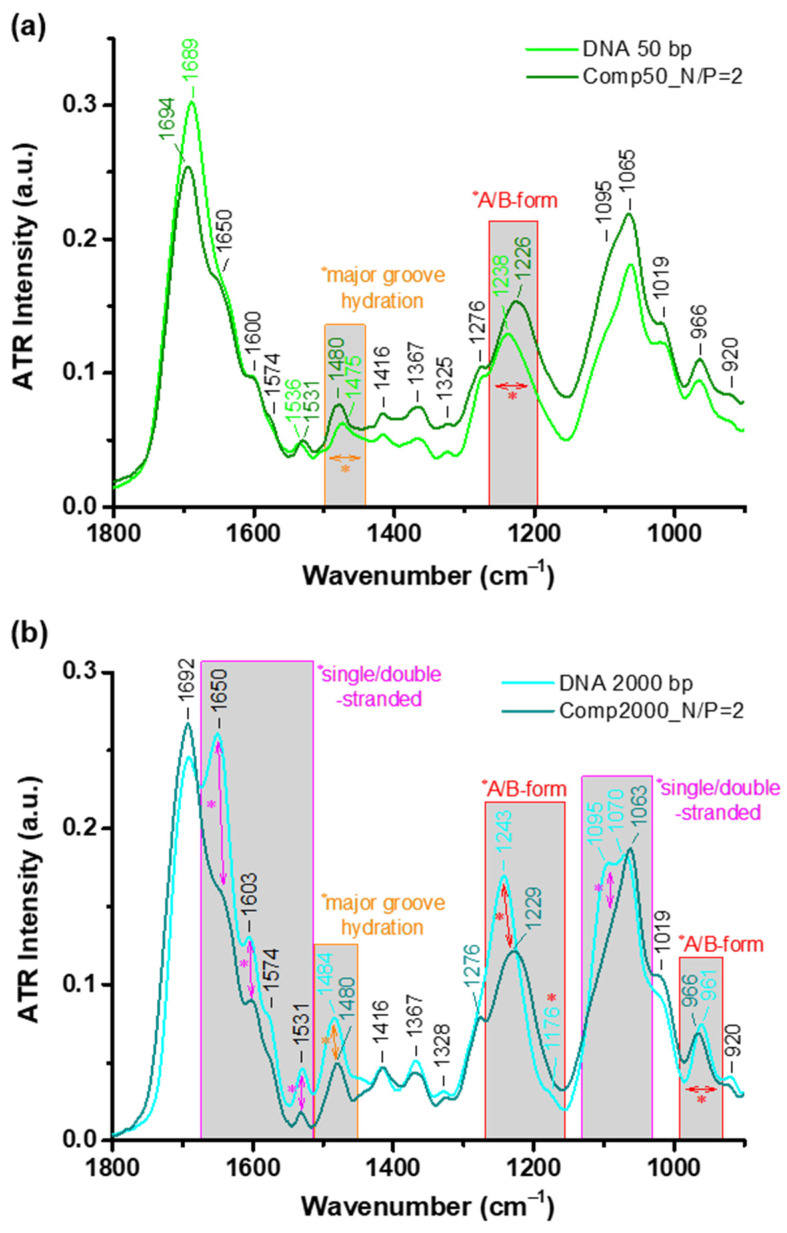
FTIR-ATR spectra in the spectral region 1800–900 cm^−1^ for the stable polyplexes formed at N/P = 2 of both (**a**) Chit-*g*-PNIPAM+DNA50 and (**b**) Chit-*g*-PNIPAM+DNA2000 systems, in comparison to the corresponding neat DNA samples (i.e., 50 or 2000 bp).

Extremely useful and detailed information concerning the conformation of the DNA molecules after their incorporation in the polyplexes can be gained through FTIR spectroscopy, which constitutes an invaluable tool for studying DNA structure [32,33,34,35,36,37,38]. To this end, FTIR-ATR spectroscopic measurements of the stable polyplexes at N/P = 2 for both Chit-*g*-PNIPAM+DNA50/2000 systems were performed by drying a small aliquot of the dispersion directly on the ATR crystal. For comparison reasons the corresponding solutions of the pure Chit-*g*-PNIPAM copolymer, along with the two DNA samples, were also measured in a similar manner. By examining the obtained spectra for the polyplexes of the two systems in contrast to the corresponding DNAs, the effect of the interaction with the graft copolymer on the structure of the DNA molecules can be derived. Figure 7 presents the FTIR-ATR spectra in the characteristic spectral region from 1800 to 900 cm^−1^, where the position of the peaks and spectral regions indicating characteristic changes are noted accordingly. Moreover, Table 2 summarizes the assignment of the discerned peaks, along with the interpretation of the observed changes according to the literature [32,33,34]. It should be noted that the spectral contribution of the Chit-*g*-PNIPAM copolymer has been appropriately subtracted from the spectra of the polyplexes.

Upon inspection of the spectra in Figure 7a, it is rather clear that for the DNA50 sample there are only a few differences observed between the neat molecules and those incorporated into the polyplexes. More specifically, the position of the peak attributed to the asymmetric ${PO}_{2}^{-}$ stretching (i.e., 1225–1245 cm^−1^) is the main marker for distinguishing between the A- and B-form of the DNA helical conformation. Thus, in this case it seems that the neat short DNA chains adopt the A-form, while the ones incorporated into the polyplexes are in the B-form. This difference in conformation is directly correlated to the degree of hydration of the DNA molecule, since fully hydrated chains are in the B-form [33,34]. These changes in the hydration degree of the DNA are also evidenced through shifts (like the one seen in Figure 7a) or intensity variations of the peak located in the 1475–1480 cm^−1^ region, which is related to the conformation of the major groove [32]. Apparently, due to the specific measuring protocol that entails the drying of the liquid sample on the ATR crystal, the neat DNA chains become dehydrated, while the ones incorporated into the polyplexes are protected against dehydration, as a result of the presence of the graft copolymer. In other words, the interaction with the Chit-*g*-PNIPAM copolymer protects the innate conformation and hydration of the short DNA chains.

As far as the long DNA sample is concerned, there are even more spectral markers, suggesting that the neat DNA2000 molecule adopts the A-form of the helical structure, i.e., the peaks discerned at 1243, 1176, and 961 cm^−1^. The additional spectral characteristics could be attributed to the longer length of the sample that probably enables a more well-defined conformation. On the other hand, the DNA2000 chains within the polyplexes are mainly in the B-form (according to the position of the corresponding peaks), indicating a more hydrated structure, a fact that is also evidenced by the variations in intensity and position of the 1480/1484 cm^−1^ band. Furthermore, the differences in intensity of the peaks located at about 1650, 1603, 1531, and 1095 cm^−1^ observed in this case suggest some degree of single- to double-strand transition between the neat DNA2000 chains and the ones incorporated into the polyplexes [32,33,34]. One possible explanation for this observation is that as the longer DNA chains are bound to additional conformational constraints, the neat chains are more susceptible to structural changes upon drying. Nevertheless, the interaction of the long DNA with the graft copolymer preserves it not only from dehydration but also from possible structural changes upon drying, thus strengthening its native structure.

## 4. Conclusions

The present experimental study focuses on the interaction between a custom-made Chit-*g*-PNIPAM copolymer and two DNA molecules of different lengths, as well as on the physicochemical properties of the formed polyplexes. The properties of the resulting polyplexes’ aqueous dispersions for both systems were investigated by means of dynamic and electrophoretic light scattering (DLS and ELS) methods. The overall physicochemical characteristics of the formed polyplexes in regard to their mass, size, charge, structure, and stability proved to be a sophisticated combination of the ratio between the two macromolecular components, the length of the DNA molecule, and the innate conformation of the Chit-*g*-PNIPAM copolymer. Moreover, the presence of the PNIPAM side chains imparts thermoresponsive behavior to the polyplexes, thus allowing for additional functionality, with fully reversible conformational changes of the already formed polyplexes taking place upon the increase in temperature above 35 °C. The stability of the polyplexes against the increase in the ionic strength or upon interacting with biological fluids like FBS was also examined and they were found remarkably resistant to both destabilizing factors. Furthermore, the binding affinity between the copolymer and the DNA chains was monitored through the fluorescence quenching of appropriate probes (i.e., ethidium bromide), and it was shown that it is greatly influenced by the length of the DNA molecule as expected, in the sense that shorter chains are more amenable to complexation due to their rod-like conformation and thus more easily incorporated in the formed polyplexes. Finally, FTIR spectroscopic measurements were conducted in order to probe the structure of the DNA chains incorporated into the polyplexes and the obtained spectra revealed that the Chit-*g*-PNIPAM copolymer protects the native conformation of both the short and the long DNA molecules. In general, studies like this one showcase the complexity of such systems and ways to characterize them from a physicochemical point of view, while in parallel demonstrating the number of parameters one has to take into consideration, when designing potential gene carrier systems intended for biological applications.

## Figures and Tables

**Table 1:** The sample codes, the concentrations of Chit-*g*-PNIPAM and DNA samples (DNA50 or 2000) in the dispersion, the total concentration, and the estimated amino to phosphate group molar ratio, N/P, for the Chit-*g*-PNIPAM+DNA50/2000 polyplexes.

Sample Code	C_Chit-*g*-PNIPAM_ (μg/mL)	C_DNA50/2000_ (μg/mL)	C_Total_ (μg/mL)	N/P
CompX_N/P = Y *	20	7	27	4
		14	34	2
		28	48	1
		56	76	0.5

* Where X stands for the number of bp of the DNA samples (50 or 2000), and Y for the N/P value.

**Table 2:** The position of the peaks, their vibrational assignments, and the interpretation of characteristic changes, observed in the FTIR-ATR spectra in the spectral region 1800–900 cm^−1^ for the stable polyplexes formed at N/P = 2 of both Chit-*g*-PNIPAM+DNA50/2000 systems.

Peak Position		Assignment		Characteristic Changes Interpretation
DNA50	DNA2000			
1694/1689	1692	C=O stretching:	Of thymine	Peaks at 1650, 1603, and 1531 cm^−1^: single/double-stranded markers
1650			Of cytosine	
1600	1603	In-plane C=N ring vibrations:	Of guanine	
1574			Of adenine	
1536/1531	1531		Of cytosine	
1480/1475	1484/1480		Of adenine and N7C8H bending of adenine/guanine	Major groove hydration change
1416		Deoxyribose:	C3′-endo	A-form marker
1367			C2′/C3′-endo	
1325	1328		C2′-endo/anti	
1276			CN3H bending of thymine	
1238/1226	1243/1229	Backbone:	${PO}_{2}^{-}$ asymmetric stretching	Main A/B-form markers
–	1176		C3′-endo/anti	A-form marker
1095			${PO}_{2}^{-}$ symmetric stretching	Single/double-stranded marker
1065	1070/1063		C–O stretching of furanose	
1019			furanose vibration	
966	966/961		C–C stretching	A/B-form markers
920			ribose ring vibration	

## Data Availability

Data are contained within the article and Supplementary Materials.

## Supplementary Materials

The following supporting information can be downloaded at: https://www.mdpi.com/article/10.3390/polym16213101/s1, Figure S1: The influence of temperature for the stable polyplexes formed at N/P = 1 of the Chit-*g*-PNIPAM+DNA50 and N/P = 0.5 of the Chit-*g*-PNIPAM+DNA2000 systems; Figure S2: The influence of temperature for the pure Chit-*g*-PNIPAM copolymer; Figure S3: The influence of ionic strength for the pure Chit-*g*-PNIPAM copolymer; Table S1: The *R*_h_ values of the different peaks, for the polyplexes at N/P = 4 of both Chit-*g*-PNIPAM+DNA50/2000 systems, after mixing with FBS solutions; Figure S4: The influence of interaction with FBS for the pure Chit-*g*-PNIPAM copolymer; Figure S5: DLS results for the two different content FBS solutions; Figure S6: EtBr fluorescence quenching in the polyplexes formed between the Chit-*g*-PNIPAM copolymer and the two DNA samples.
