# Supplementary material for: Coassembly of a Hybrid Synthetic–Biological Chitosan-g-Poly(N-isopropylacrylamide) Copolymer with DNAs of Different Lengths
Source: Polymers (Basel). 2024 Nov 4;16(21):3101. doi: 10.3390/polym16213101 (PMC11548708; doi:10.3390/polym16213101)
Supplement: Supplementary file 1 [file polymers-16-03101-s001.zip › polymers-3260099-supplementary.pdf]

Supplementary Materials

Coassembly of a Hybrid Synthetic–Biological Chitosan-*g*-Poly(*N*-isopropylacrylamide) Copolymer with DNAs of Different Lengths

Maria Karayianni [1], Elena-Daniela Lotos [1], Marcela Mihai [1,*] and Stergios Pispas [1,2,*]

[1] Petru Poni Institute of Macromolecular Chemistry, 41A Grigore Ghica Voda Alley, 700487 Iasi, Romania; m.karayianni@icmpp.ro (M.K.); daniela.lotos@icmpp.ro (E.-D.L.)
[2] Theoretical and Physical Chemistry Institute, National Hellenic Research Foundation, 48 Vassileos Constantinou Ave., 116 35 Athens, Greece
* Correspondence: marcela.mihai@icmpp.ro (M.M.); pispas@eie.gr (S.P.)

Temperature Response of the Chit-*g*-PNIPAM+DNA Polyplexes

Additional temperature dependent DLS measurements were performed on the Chit-*g*-PNIPAM+DNA polyplex dispersions corresponding to N/P = 1 for the DNA50 system and N/P = 0.5 for the DNA2000 one, as well as for the pure Chit-*g*-PNIPAM copolymer stock solution (0.1 mg/mL in 0.5% *v*/*v* acetic acid) and the obtained results in regard to the increase of the scattered intensity (i.e., the initial values have been normalized to zero for clarity), and the R_h values of the different peaks discerned in the corresponding size distribution functions (SDFs), as a function of temperature (from 25 to 45 °C with a 5 °C increment), are shown in Figure S1 and S2, respectively. It should be noted that after the heating up to 45 °C the samples were brought back to 25 °C and measured again (so as to examine the reversibility of the system), with these measurements being marked as AH (i.e., "after heating") and the corresponding values being separated by a dashed line.

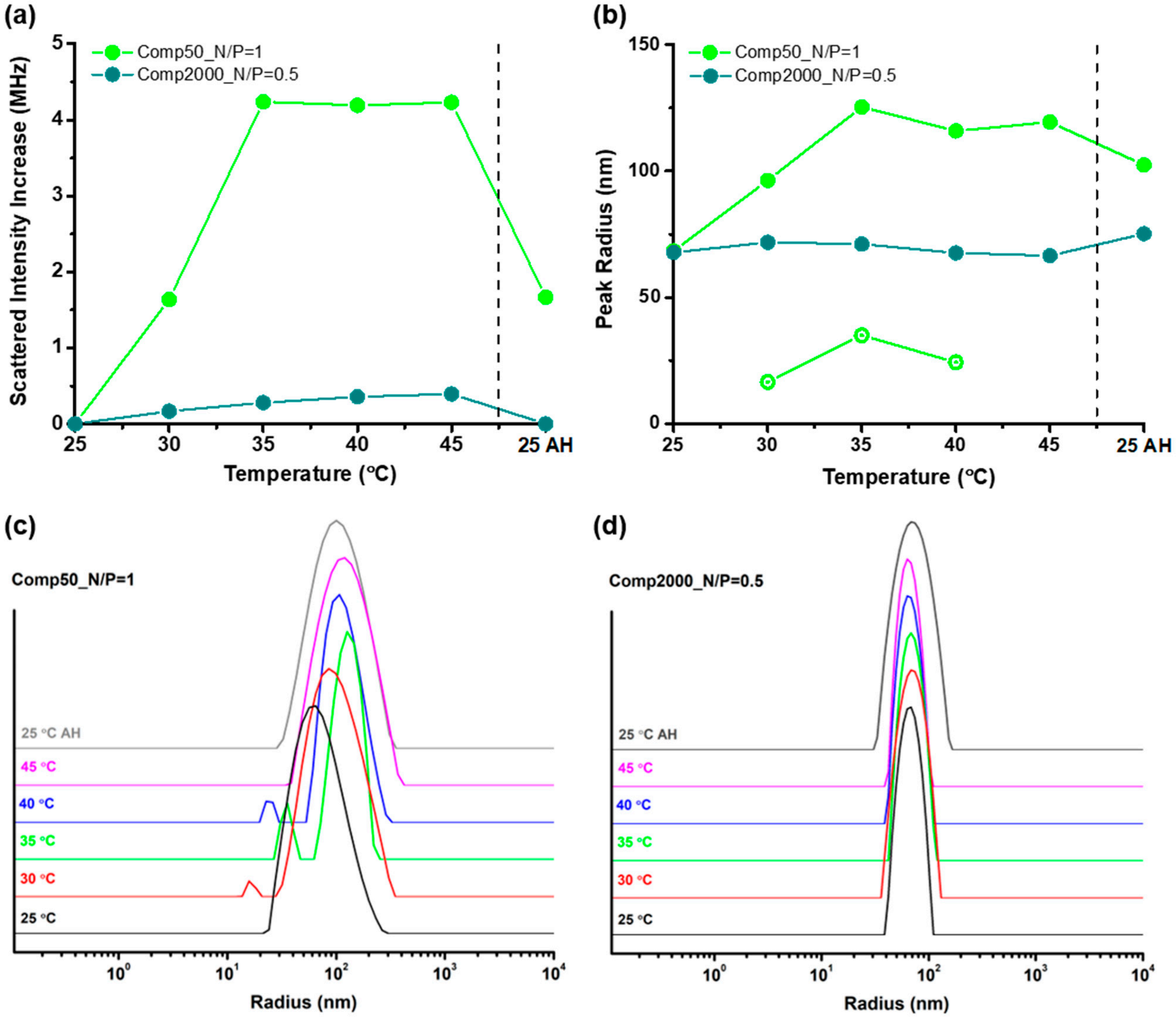

Figure S1. The influence of temperature on (**a**) the scattered intensity, and (**b**) the hydrodynamic radius derived from the peaks (open and closed symbols denote different peaks according to size) of the corresponding (**c, d**) SDFs, for the stable polyplexes formed at (**c**) N/P = 1 of the Chit-*g*-PNIPAM+DNA50 and (**d**) N/P = 0.5 of the Chit-*g*-PNIPAM+DNA2000 systems, respectively.

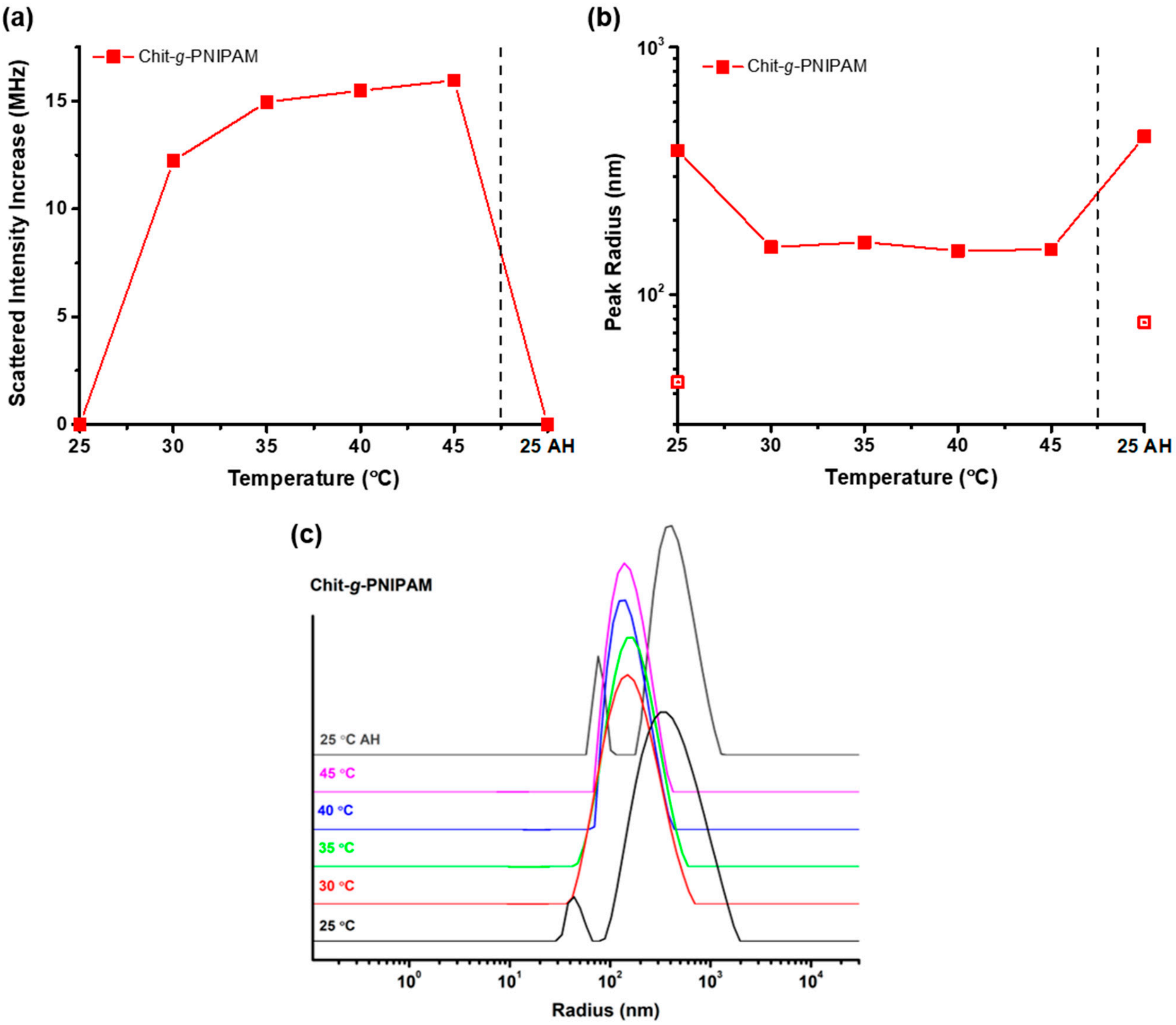

Figure S2. The influence of temperature on (**a**) the scattered intensity, and (**b**) the hydrodynamic radius derived from the peaks (open and closed symbols denote different peaks according to size) of the corresponding (**c**) SDFs, for the pure Chit-*g*-PNIPAM copolymer.

In accordance to the observations made for the stable polyplexes at N/P = 4 of both Chit-*g*-PNIPAM+DNA50/2000 systems, the polyplexes formed with the short DNA sample at N/P = 1 seem to undergo secondary aggregation upon heating, as evidenced by the observed intense increase of the scattered intensity and the noticeable increase of their corresponding size. On the other hand, the polyplexes of the DNA2000 sample corresponding to N/P = 0.5 seem practically unaffected by the increase of temperature (apart from a small increase of the scattered intensity). Of course, the respective behaviors are a direct consequence of the internal structure of the corresponding polyplexes and the specific conformations of individual components in them (especially the degree of conformational restrictions to the PNIPAM chains which are the

thermoresponsive part of the systems and the state of interactions with the aqueous environment). Specifically, for the DNA50 sample the polyplexes are already characterized by a rather compact/dense structure, so upon heating the increase of the hydrophobic interactions due to PNIPAM hinders their solubility leading to further aggregation. In the case of the DNA2000 sample, the excess of the DNA chains in combination to their increased length seem to impart additional stability to the formed polyplexes, even against the increase of temperature, thus compensating for the occurring increase of hydrophobicity.

Ionic Strength Effect on the Chit-*g*-PNIPAM Copolymer

In order to examine the behaviour of the Chit-*g*-PNIPAM graft copolymer against the increase of the ionic strength, appropriate titrations (by addition of specific aliquots of a 1 M NaCl solution) were performed and followed by means of DLS measurements. The obtained results in regard to the change of the scattered intensity (i.e., the initial values have been normalized to one for clarity), and the R_h values of the different peaks from the corresponding SDFs, for the Chit-*g*-PNIPAM stock solution (0.1 mg/mL in 0.5% *v*/*v* acetic acid), as a function of the ionic strength are presented in Figure S3. As it can be seen, upon the initial addition of salt (0.05 M) in the copolymer solution a significant decrease of the scattered intensity is observed, accompanied by a similar decrease of the size of the larger population. These transitions most probably indicate the dissociation of the multi-chain aggregates of the Chit-*g*-PNIPAM copolymer due to the reduction of the hydrophobic interactions. In other words, the solubility of the graft copolymer is enhanced at low ionic strength values, in accordance to the salting-in effect. Subsequent increase of the salt content leads to a gradual increase of the scattered intensity up to 0.2 M, while for ionic strength values above 0.33 M an abrupt drastic increase of scattered intensity occurs, without equivalent changes of the size (apart from the fact that only one peak is discerned), signifying the formation of compact/dense structures. Apparently, at higher ionic strength values the solubility of the Chit-*g*-PNIPAM copolymer worsens drastically, leading to aggregation as the salting-out effect takes over, as a consequence of the increased hydrophobic interactions and the extensive charge screening.

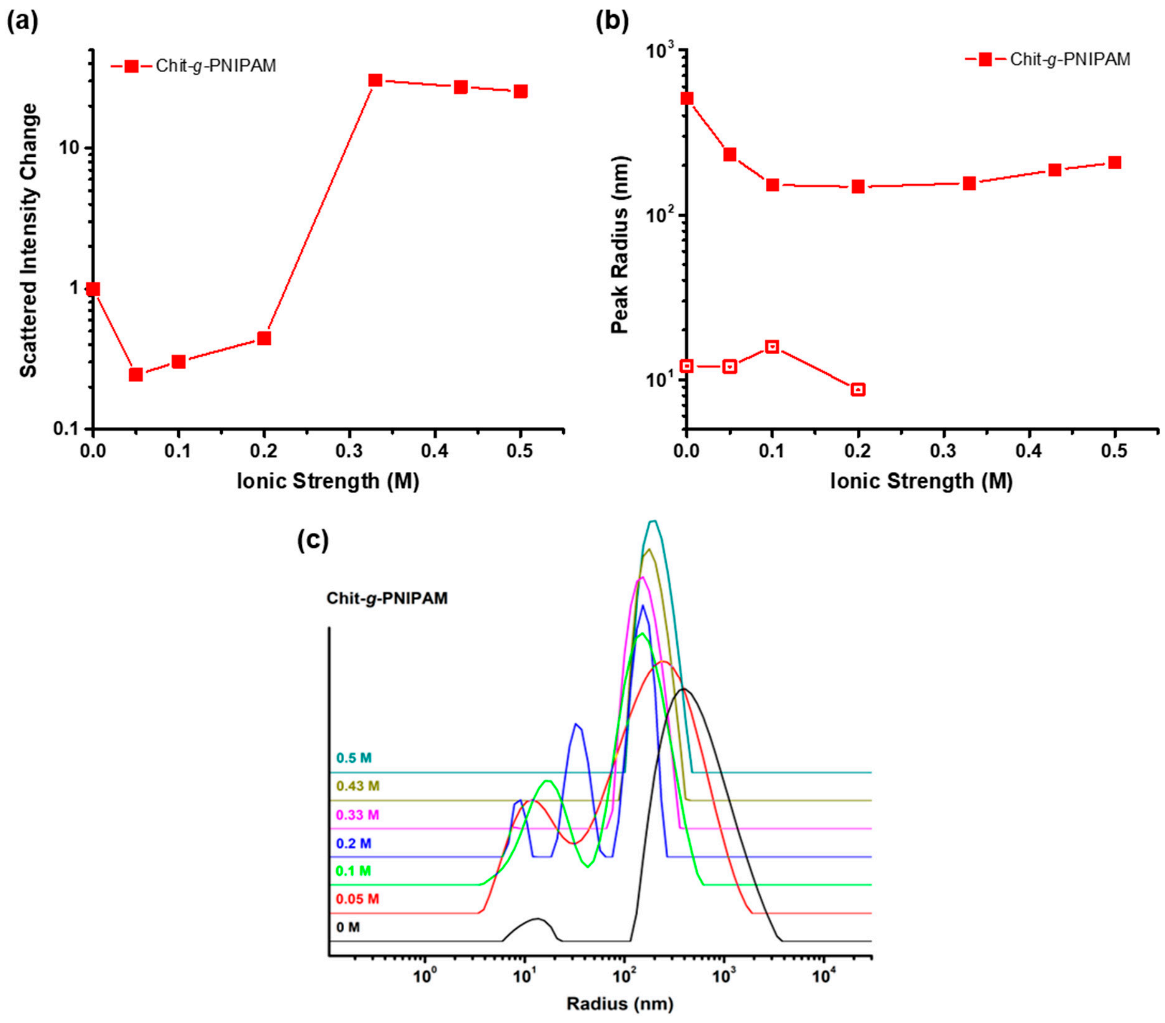

Figure S3. The influence of ionic strength on (**a**) the scattered intensity, and (**b**) the hydrodynamic radius derived from the peaks (open and closed symbols denote different peaks according to size) of the corresponding (**c**) SDFs, for the pure Chit-*g*-PNIPAM copolymer.

Changes in R_h Values of the Polyplexes Upon Interaction with FBS

In order to better elucidate the observed changes in the R_h values of the different peaks (derived from the corresponding SDFs) shown in Figure 5b, since there is some overlapping, the obtained values are also presented in the following Table S1. Note that the small peaks with sizes below 20 nm seen in the SDFs of Figure 5c and d (i.e., R_{h1} and R_{h2}) are attributed to FBS (see Figure S5) and have been excluded from Table S1 for clarity reasons.

Table S1. The R_h values of the different peaks from the corresponding SDFs, for the polyplexes at N/P = 4 of both Chit-*g*-PNIPAM+DNA50/2000 systems, after mixing with FBS solutions of different content (10 and 50% *v*/*v*).

Sample Code	FBS Content (% *v*/*v*)*	R_{h3} (nm)	R_{h4} (nm)
Comp50_N/P=4_25 °C	0	24.5	426.3
	10		477.4
	50	52.1	1150
Comp50_N/P=4_37 °C	10	94.5	689.3
	50		1215
Comp2000_N/P=4_25 °C	0	54.1	
	10	117.4	453.1
	50	52.2	1019
Comp2000_N/P=4_37 °C	10	62.3	467
	50	344.8	1309

* Where 0 FBS content denotes the initial state

Stability of the Chit-*g*-PNIPAM Copolymer in FBS

The stability of the pure Chit-*g*-PNIPAM copolymer against biological media like FBS was also investigated, following a similar procedure to the one performed for the polyplex dispersions. Figure S4 shows the obtained DLS results in regard to the increase of the scattered intensity (i.e., the initial values have been shifted to zero for clarity), and the R_h values of the different peaks from the corresponding SDFs, for the Chit-*g*-PNIPAM stock solution, after mixing with FBS solutions of different content (10 and 50% *v*/*v*). Measurements were performed first at 25 °C and then at 37 °C after 30 min incubation at the final temperature. It should be noted that the values at zero FBS content denote the initial state of the copolymer solution. Additionally, the small peaks with sizes below 20 nm (i.e., open symbols in Figure S4b) discerned in the SDFs are attributed to FBS as seen in Figure S5.

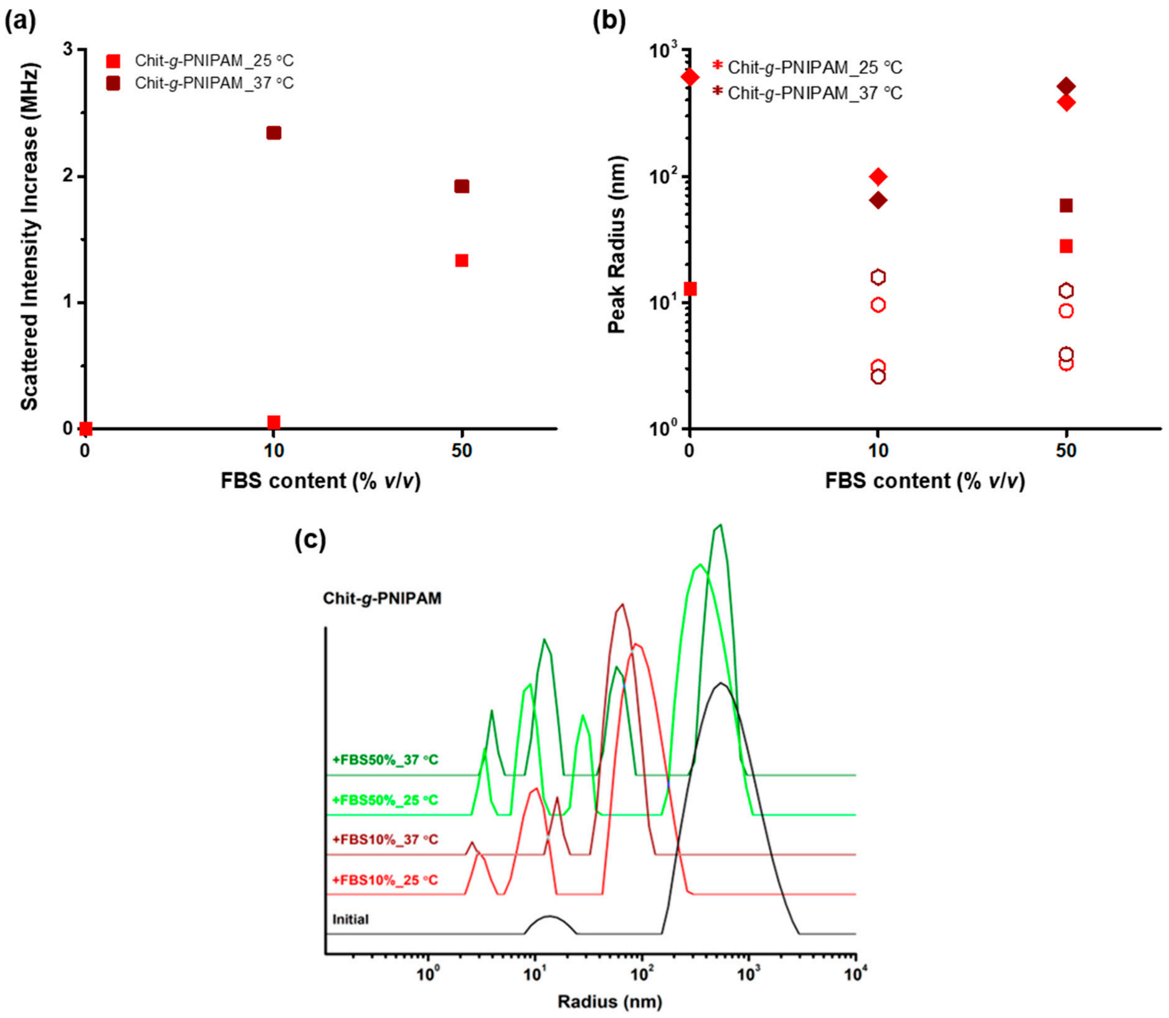

Figure S4. The influence of interaction with FBS on (**a**) the scattered intensity, and (**b**) the hydrodynamic radius derived from the peaks (various symbols denote different peaks according to size, with open ones attributed to FBS) of the corresponding (**c**) SDFs, for the pure Chit-*g*-PNIPAM copolymer and the two FBS solutions of different content (10 and 50% *v*/*v*).

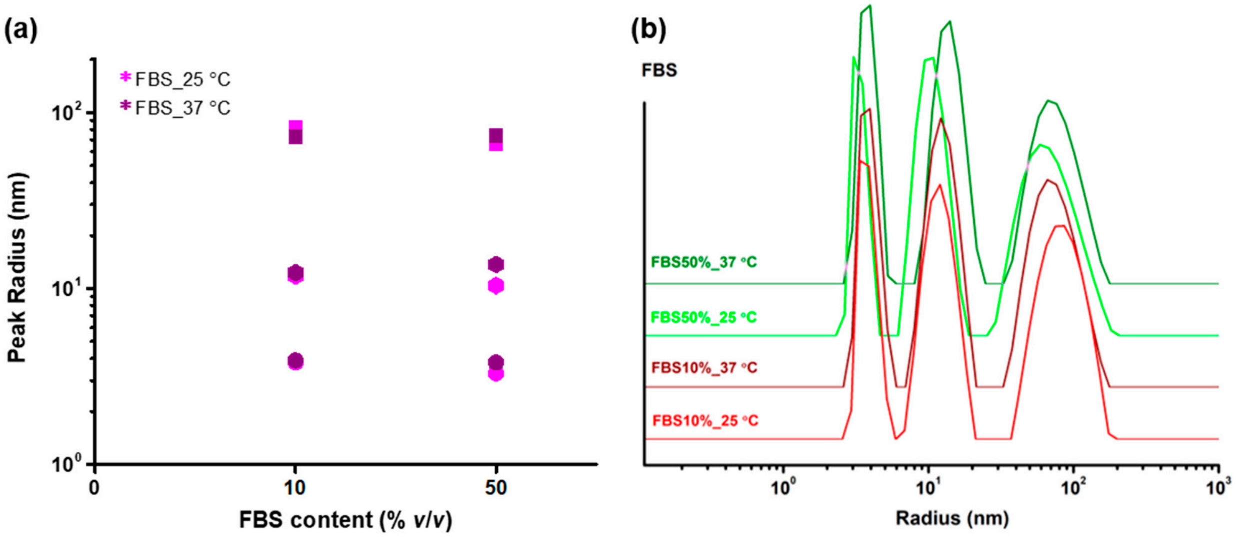

Figure S5. DLS results in regard to (**a**) the hydrodynamic radius values derived from the peaks (various symbols denote different peaks according to size) of the corresponding (**b**) SDFs, for the two different content FBS solutions (i.e., 10 and 50% *v*/*v* in PBS).

As seen in Figure S4, upon mixing with the low protein content FBS solution, the scattered intensity remains practically the same, while at the same time a significant decrease of the size of the larger population in solution is observed. It appears that the binding of the FBS components causes the shrinking of the multi-chain aggregates of the Chit-*g*-PNIPAM copolymer, due to the occurring charge screening or additional conformational changes due to protein binding. This fact also favors the hydrophobic interactions of the system, which become even more prominent after heating at 37 °C, causing further collapse of the structure of the mixed particles into more compact structures, as evidenced by the significant increase of the scattered intensity. On the other hand, at high FBS content the mass of the particles in solution is increased, even though their corresponding size is similar to the original of the copolymer multi-chain aggregates. Most probably, under these conditions the aggregates of the copolymer interact with a large number of FBS proteins/enzymes, thus forming mixed particles bigger in size than those formed at lower FBS content. Moreover, they seem to have a rather compact/dense structure, and therefore are less prone to structural rearrangements, since the increase of temperature does not cause any significant changes of either their mass or size. Overall, the Chit-*g*-PNIPAM graft copolymer proves to be stable against biological fluids.

EtBr Fluorescence Quenching

The acquired emission spectra following the fluorescence quenching of EtBr during the titration of both EtBr-labeled DNA samples (i.e., 50 and 2000 bp) with the Chit-*g*-PNIPAM copolymer, in a range of N/P ratios from 0 (initial labeled DNA solution) up to 6, are presented in Figure S6.

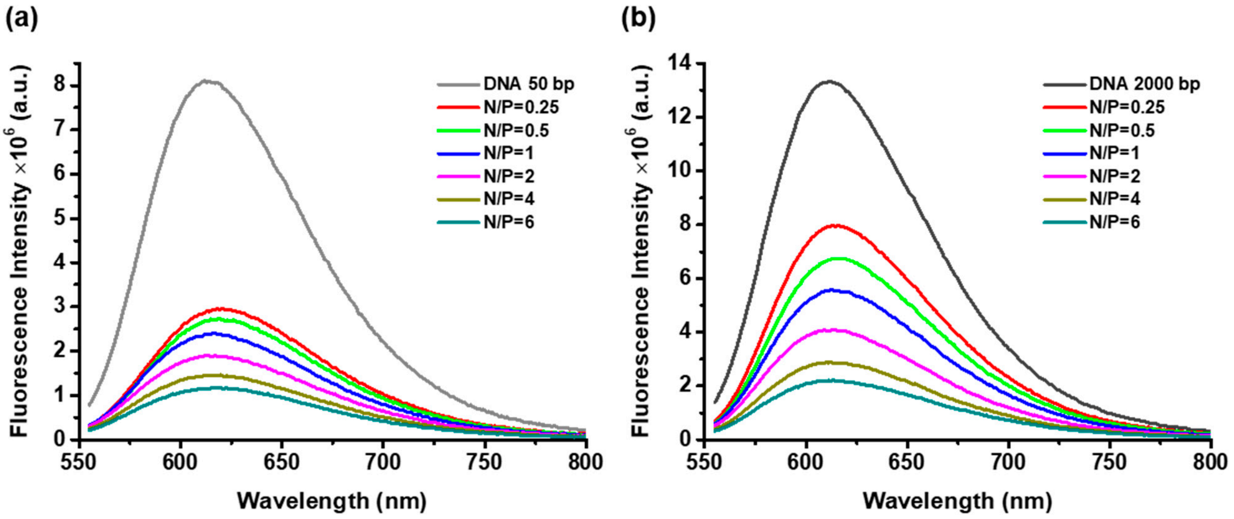

Figure S6. EtBr fluorescence quenching in the polyplexes formed between the Chit-*g*-PNIPAM copolymer and the (**a**) DNA 50 bp or (**b**) DNA 2000 bp sample.
